# Aberrantly hypermethylated tumor suppressor genes were identified in oral squamous cell carcinoma (OSCC)

**DOI:** 10.1186/s13148-019-0715-0

**Published:** 2019-08-12

**Authors:** Soo Yeon Kim, Yu Kyeong Han, Jae Min Song, Chang Hun Lee, Keunsoo Kang, Joo Mi Yi, Hae Ryoun Park

**Affiliations:** 1 Department of Oral Pathology, School of Dentistry, Pusan National University, Yangsan, 50612 Gyeongsangnam-do Republic of Korea; 2 Department of Microbiology and Immunology, College of Medicine, Inje University, Busan, 47392 Republic of Korea; 3 Department of Oral and Maxillofacial Surgery, Pusan National University, Yangsan, 50612 Gyeongsangnam-do Republic of Korea; 4 Dental and Life Science Institute, School of Dentistry, Pusan National University, Yangsan, 50612 Gyeongsangnam-do Republic of Korea; 5 Department of Pathology, School of Medicine, Pusan National University, Busan, 49241 Republic of Korea; 6 Department of Microbiology, Dankook University, Cheonan, 31116 Republic of Korea

**Keywords:** DNA methylation, Tumor suppressor gene, Oral squamous cell carcinoma, Gene silencing, Prognosis biomarker

## Abstract

**Background:**

Oral squamous cell carcinoma (OSCC) is a genetic and epigenetic disease. There is growing evidence to suggest that environmental factors due to epigenetic changes can be involved in the OSCC pathogenesis. Although tumor suppressor genes (TSGs) are commonly inactivated by promoter hypermethylation in human cancers, the epigenetic changes and the mechanism of TSGs in human OSCC remain unclear. We therefore assessed the methylation status of the TSGs, which are associated with epigenetic silencing in human cancers, OSCC cell lines, primary tumors, and normal oral mucosa.

**Results:**

We used 14 TSGs that were originally identified in colon cancer to investigate the aberrant hypermethylation of these genes associated with transcriptional silencing in 10 OSCC cell lines. We found three TSGs, *TFPI2*, *SOX17*, and *GATA4*, that are robustly hypermethylated and are associated with transcriptional silencing in OSCC cell lines. The re-expression of the three genes was induced by 5-aza-2′-deoxycytidine (5-aza-dC) in cells in which these genes were not expressed or had a lack of expression. In 33 cases of primary OSCC tumors, promoter hypermethylation was detected for the *TFPI2*, *SOX17*, and *GATA4* genes at (32/33) 97%, (22/33) 67%, and (11/33) 33%, respectively. Eleven normal oral mucosa samples showed no promoter hypermethylation for all three genes, which suggests that this promoter hypermethylation is cancer-specific. Bisulfite sequencing analysis confirmed the cancer-specific methylation of the *TFPI2*, *SOX17*, and *GATA4* promoters in the OSCC cell lines and tumors but not in the normal oral mucosa samples. More importantly, the methylation status of *TFPI2*, *GATA4*, and *SOX17* was significantly associated with OSCC patients’ overall survival through TCGA DNA methylation database.

**Conclusions:**

We identified that *TFPI2*, *SOX17*, and *GATA4* are frequently hypermethylated in human OSCC cells in a cancer-specific manner and that the transcriptional expression of these genes is regulated by promoter hypermethylation in OSCC. Our results highlight the great potential used as a synergistic biomarker set to improve the prognosis and therapeutic treatment for patients with OSCC.

**Electronic supplementary material:**

The online version of this article (10.1186/s13148-019-0715-0) contains supplementary material, which is available to authorized users.

## Background

Epigenetic modification, including DNA methylation and many types of histone modifications, is responsible for the altered gene expression patterns that allow for specific phenotypes [1]. DNA methylation is the primary and most studied epigenetic modification [1] and plays an important role in normal mammalian development, but aberrant methylation patterns are correlated with several differentiation-related diseases, including many types of human cancers. Gene promoter hypermethylation in cancer can silence gene expression and regulate biological processes, especially tumor suppressor genes (TSGs), which have an important role in cancer initiation, progression, and metastasis [2]. Aberrant DNA methylation is a common phenomenon in malignancies, and the methylation profiles are altered in various tumors, which might be associated with clinical outcomes [1].

We and others have reported that the transcriptional silencing of tumor suppressor candidate genes is regulated by promoter hypermethylation in colorectal cancer [3, 4]. High-throughput genome-wide methylation studies offer a sophisticated strategy to understand the significance of DNA methylation and its impact on gene regulation [3, 5].

Numerous studies have used these well-known tumor suppressor candidate genes in other cancer types, such as lung, breast, and pancreatic cancer types [4, 6]. However, there is a lack of studies on the DNA methylation profiles of these TSGs in oral squamous carcinoma cancer (OSCC). Very recent findings suggest that the oral microbiota could contribute to CRC development, implying that the profile of epigenetic changes may be shared between two different cancer types [7, 8].

Oral squamous cell carcinoma (OSCC), the most prevalent type of head and neck SCC (HNSCC), typically behaves in an aggressive manner, frequently leading to local invasion and early lymph node metastasis [9]. The overall 5-year survival rate for OSCC is approximately 60% [10] and shows only modest improvement over the past two decades, despite considerable improvements in the treatment of OSCC [11, 12]. In the last decade, there has been a lack of understanding of OSCC carcinogenesis, progression, and metastasis in terms of epigenetic regulation and its clinical application. Therefore, the identification of molecular changes for the oncogenes or TSGs associated with OSCC will help improve diagnosis predictions and early treatment [13, 14].

In this study, we investigated transcriptional silencing by the promoter hypermethylation of 18 tumor suppressor candidate genes (*MGMT*, *sFRP2*, *HIC1*, *sFRP4*, *Timp3*, *sFRP5*, *TFPI2*, *p16*, *sFRP1*, *E-cad*, *SOX17*, *GATA4*, *GATA5*, *p14*, *FBN2*, *p15*, and *TCERG1L*) in 10 OSCC cell lines and a small set of OSCC primary tumor samples (*n* = 33). Remarkably, we found that most of the genes are silenced by promoter hypermethylation in OSCC cell lines. Among these genes, *TFPI2*, *SOX17*, and *GATA4* were frequently hypermethylated in both OSCC and oral cancer patient samples in a cancer-specific manner, suggesting that these genes may play a role as tumor suppressors in OSCC.

## Results

### Identification of aberrantly hypermethylated tumor suppressor candidates in oral squamous cell carcinoma (OSCC)

**Fig. 1:**
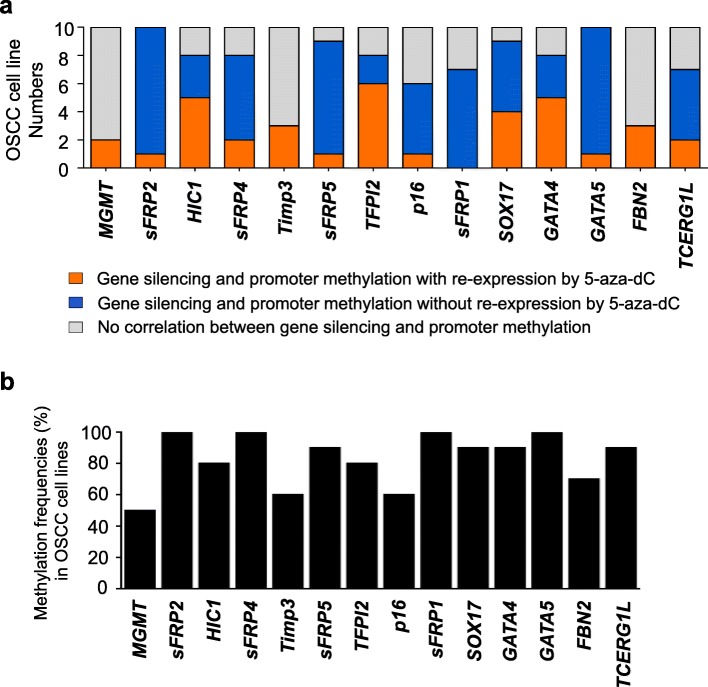
Correlation between transcriptional expression and the promoter hypermethylation of tumor suppressor genes in OSCC cell lines. **a** Summarized results based on the RT-PCR and MSP results of 14 tumor suppressor genes in 10 OSCC cell lines (Ca9-22, HSC4, OSC20, SAS, SCC25, HN22, YD10B, YD38, YD9, and HSC3). The correlation between transcriptional expression and promoter hypermethylation was categorized by the following three criteria: (1) genes are silenced by promoter hypermethylation with re-expression by 5-aza-dC treatment (indicated in yellow), (2) genes are silenced by promoter hypermethylation without re-expression by 5-aza-dC treatment (indicated in blue), and (3) genes are not correlated between gene expression and promoter methylation (indicated in gray). **b** Methylation frequencies of TSGs in 10 OSCC cell lines by MSP analysis

In this study, we investigated transcriptional silencing by the promoter hypermethylation of 14 well-known tumor suppressor candidate genes (*MGMT*, *sFRP2*, *sFRP1*, *HIC1*, *sFRP4*, *Timp3*, *sFRP5*, *TFPI2*, *p16*, *SOX17*, *GATA4*, *GATA5*, *FBN2*, and *TCERG1L*) in OSCC cell lines. These genes were originally identified from comprehensive studies of colon cancer as tumor suppressor candidate genes, and their transcriptional silencing is regulated by promoter hypermethylation in many other cancer types [4]. First, we examined whether the expression of these genes is regulated by promoter methylation in 10 OSCC cell lines (Ca9-22, HSC4, OSC20, SAS, SCC25, HN22, YD10B, YD38, YD9, and HSC3) by reverse transcriptase polymerase chain reaction (RT-PCR). We treated the OSCC cell lines with the demethylation agent 5-aza-2′-deoxycytidine (5-aza-dC) to determine whether these TSGs are re-expressed after 5-aza-dC treatment. To correlate the transcriptional expression of TSGs with promoter hypermethylation, we assessed the methylation analysis of these TSGs in OSCC cell lines by methylation-specific PCR (MSP). We performed massive RT-PCR and MSP to identify whether these TSGs are silenced by promoter hypermethylation in OSCC. The results for transcriptional expression and promoter hypermethylation by the RT-PCR and MSP analyses of TSGs are summarized in Fig. 1a (Additional file 1: Figure S1) with the following three categories: (1) genes are silenced by promoter hypermethylation with re-expression by 5-aza-dC treatment, (2) genes are silenced by promoter hypermethylation without re-expression by 5-aza-dC treatment, and (3) gene expression is not correlated with promoter methylation. The methylation frequencies of 14 TSGs in 10 OSCC cell lines ranged from 50 to 100% (Fig. 1b), which suggests that all of the genes we tested are frequently hypermethylated in OSCC cell lines.

**Fig. 2:**
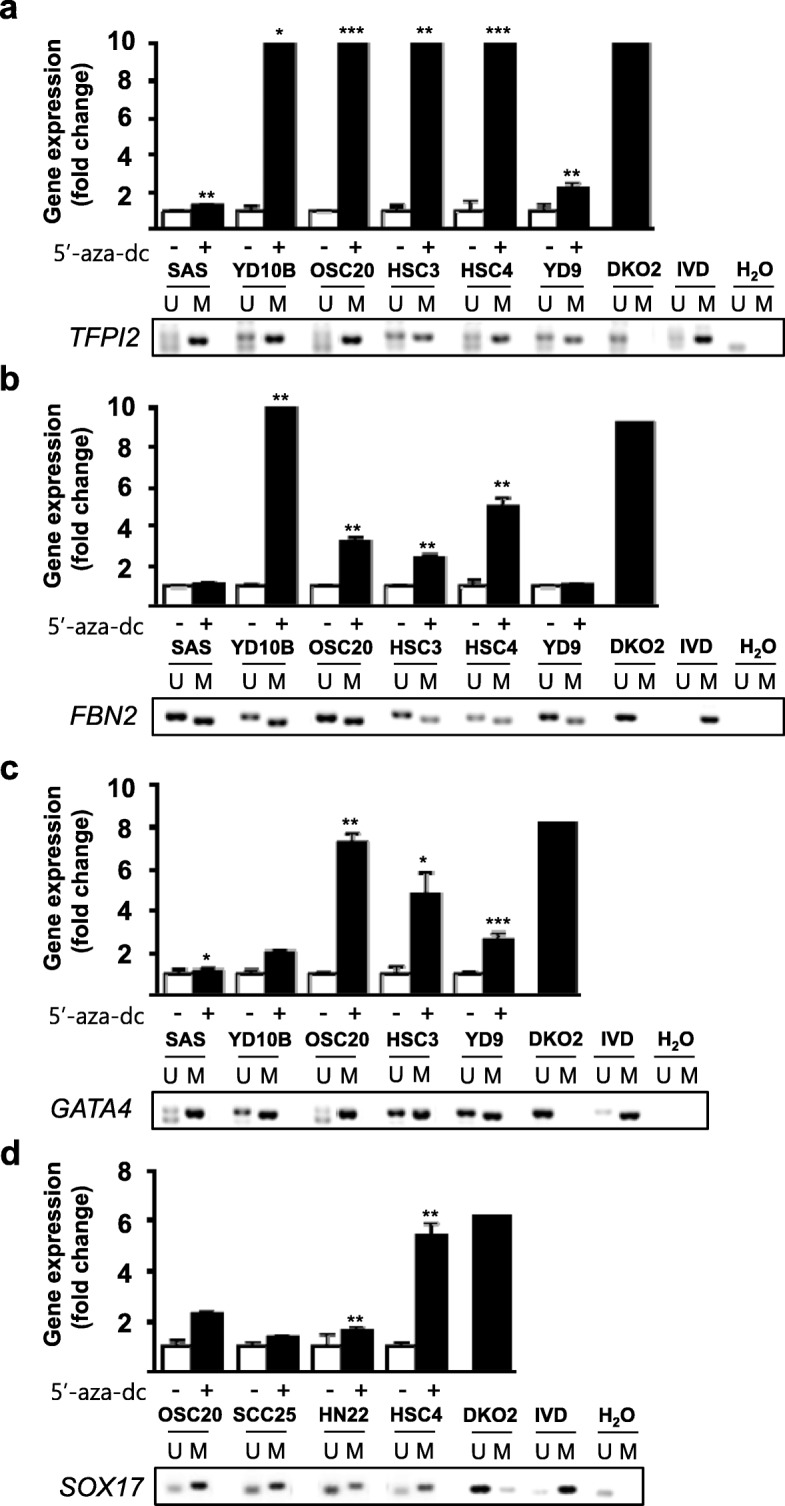
**a**–**d** Epigenetic inactivation of *TFPI2*, *SOX17*, *GATA4*, and *FBN2* in OSCC cell lines. The graph displays the expression analysis of four genes after 5-aza-dC treatment. Real-time RT-PCR analysis was carried out to assess four TSG expression levels in OSCC cell lines before and after treatment with 5 μM 5-aza-dC for 72 h. DNA methyltransferase 1 and 3b knockout HCT116 cells (DKO) were included as positive controls. Asterisk indicates a significant increase in gene expression after 5-aza-dC treatment (*p* < 0.05). The expression levels of genes were internally normalized to the expression levels of *GAPDH*, and the normalized expression for each gene before 5-aza-dC treatments was set to 1. Gel pictures describe the methylation analysis by methylation-specific PCR (MSP). PCR products recognize unmethylated (U) and methylated (M). DKO cells were used for the unmethylated control. IVD = in vitro methylated control; ddH_2_O = water control containing no DNA

**Fig. 3:**
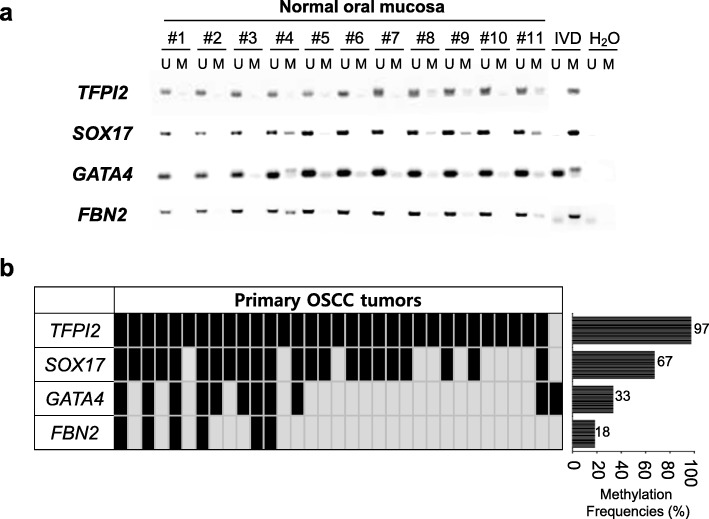
Epigenetic inactivation of *TFPI2*, *SOX17*, *GATA4*, and *FBN2* in normal oral mucosa and OSCC primary tissues. **a** Methylation status of *TFPI2*, *SOX17*, *GATA4*, and *FBN2* in normal oral mucosa (*n* = 11) and **b** the methylation frequency of the four genes in OSCC primary tissues (*n* = 33). M = methylation signal; U = unmethylated signal; IVD = in vitro methylated DNA; ddH_2_O = water control without DNA. The bar graph shows the MSP results of all primary samples

The silencing of most TSGs is regulated by promoter hypermethylation, and the genes are re-expressed by 5-aza-dC treatment. We found that the transcriptional silencing of 9 genes (*MGMT*, *HIC1*, *sFRP4*, *Timp3*, *TFPI2*, *SOX17*, *GATA4*, *FBN2*, and *TCERG1L*) out of 14 genes by promoter hypermethylation was correlated with gene re-expression by 5-aza-dC treatment in 2 or more OSCC cell lines. Nonetheless, we decided to focus on 4 TSGs (*TFPI2*, *SOX17*, *GATA4*, and *FBN2*) because we later confirmed that these genes had a cancer-specific methylation pattern in primary OSCC tumor samples (Fig. 3). We determined whether these 4 genes are re-expressed after 5-aza-dC treatment in 10 OSCC cell lines by real-time RT-PCR (Fig. 2). We postulate that these genes were re-expressed in OSCC cells after 5-aza-dC treatment, and we should detect their promoter hypermethylation in those cells. To verify that transcriptional silencing was due to promoter hypermethylation, we assessed the methylation levels of the *TFPI2*, *SOX17*, *GATA4*, and *FBN2* genes by MSP. Overall, we identified four TSGs (*TFPI2*, *SOX17*, *GATA4*, and *FBN2*) that are regulated by promoter hypermethylation associated with transcriptional silencing in OSCC cell lines.

### *TFPI2*, *SOX17*, and *GATA4* are frequently hypermethylated in OSCC cells and primary OSCC tumors in a cancer-specific manner

To validate that four TSGs were indeed regulated by promoter hypermethylation, we extended our methylation analysis for these genes in a panel of primary OSCC tumor samples (*n* = 33) and normal oral mucosa samples (*n* = 11) obtained from cancer-free individuals. The clinical characteristics of the patients are documented in Table 1. Four genes (*TFPI2*, *SOX17*, *GATA4*, and *FBN2*) satisfied the criteria of “cancer-specific methylation” with high-frequency methylation in cell lines, no/undetectable methylation in normal oral mucosa (Fig. 3a), and frequent methylation in primary OSCC tumor samples (Fig. 3b). Based on our data, we decided to eliminate *FBN2* for further experimental analyses because of the low frequency (< 20%) of methylation in primary OSCC tissue samples.

**Table 1:** Basic characteristics of the OSCC patient in this study

Characteristics	*N*
Total no. of patients	34
Age (years)	
Median (range)	67 (30–90)
Average (St. dev.)	63.41 (14.1)
Gender, *n* (%)	
Male	18 (52.9)
Female	16 (47.1)
Stage, *n* (%)	
1	8 (23.5)
2	10 (29.4)
3	4 (11.8)
4	12 (35.3)
Lymph node metastasis, *n* (%)	
Negative	21 (61.8)
Positive	10 (29.4)
Unknown	3 (8.8)
Grade, *n* (%)	
Well differentiated	25 (73.5)
Moderate differentiated	5 (14.7)
Poor differentiated	3 (8.8)
Smoking, *n* (%)	
No	28 (82.4)
Yes	6 (17.6)
Alcohol, *n* (%)	
No	23 (67.6)
Yes	11 (32.4)
Recurrence, *n* (%)	
No	25 (73.5)
Yes	9 (26.5)

**Fig. 4:**
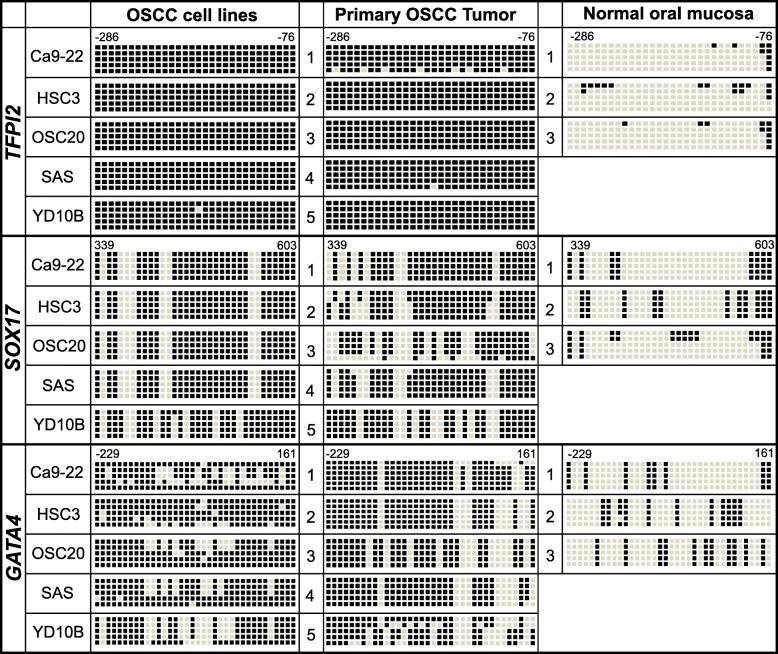
Bisulfite sequencing analyses of the CpG islands in the *TFPI2*, *SOX17*, and *GATA4* gene promoter regions. Representative bisulfite sequencing analyses were performed for all three genes in representative OSCC cell lines (Ca9-22, HSC3, OSC20, SAS, and YD10B), OSCC primary tumors (*n* = 5), and normal oral mucosa (*n* = 3). The location of CpG sites (*TFPI2*: upstream region from − 286 to − 76; *SOX17*: exon 1 region from + 339 to + 603; and *GATA4*: upstream region from − 229 to + 161) relative to the transcription start sites (TSSs) of exon 1. Each box represents a CpG dinucleotide. The black boxes represent methylated cytosines, and the white boxes represent unmethylated cytosines

We next assessed changes in DNA methylation status in the promoter region of the three genes by bisulfite genomic sequencing in representative OSCC cell lines (Ca9-22, HSC3, OSC-20, SAS, and YD10B), OSCC primary tumor samples (*n* = 5), and normal oral mucosa samples (*n* = 3) (Fig. 4). The location of promoter regions (*TFPI2*: upstream region from − 286 to − 76, 30 CpG sites; *SOX17*: region from + 339 to + 603, 34 CpG sites; and *GATA4*: upstream region from − 229 to 161, 46 CpG sites) for sequencing is relative to the transcription start sites (TSS) of exon 1. The bisulfite sequencing of five individual clones of PCR products from five OSCC cell lines revealed densely methylated CpG sites within the promoter regions of all of the clones. The methylation status of the *TFPI2* promoter region in OSCC cells and primary tissues showed that most of the CpG sites (> 99%) we sequenced were methylated compared to those in the normal oral mucosal tissues (< 11%). Both the *SOX17* and *GATA4* genes in OSCC cells and primary tissues (59–77% for *SOX17* and 71–91% for *GATA4*) showed relatively dense methylation than those in normal tissues (< 32% for *SOX17* and< 23% for *GATA4*). Accordingly, these bisulfite sequencing data confirm the complete methylation of the three gene promoters in OSCC cell lines, primary tumor tissues, and normal oral mucosal samples determined by MSP (Fig. 2), but they also explain the lack of these three gene expression results in OSCC cell lines obtained by quantitative RT-PCR analysis. Notably, all three genes were significantly hypermethylated in primary OSCC tumors compared to those in the normal tissues. Overall, these results strongly suggest that the *TFPI2*, *SOX17*, and *GATA4* genes showed increasing DNA methylation levels in primary OSCC tumors compared with the DNA methylation levels in normal oral tissues.

### *TFPI2*, *SOX17*, and *GATA4* protein expression is downregulated in primary OSCC tumor tissues

**Fig. 5:**
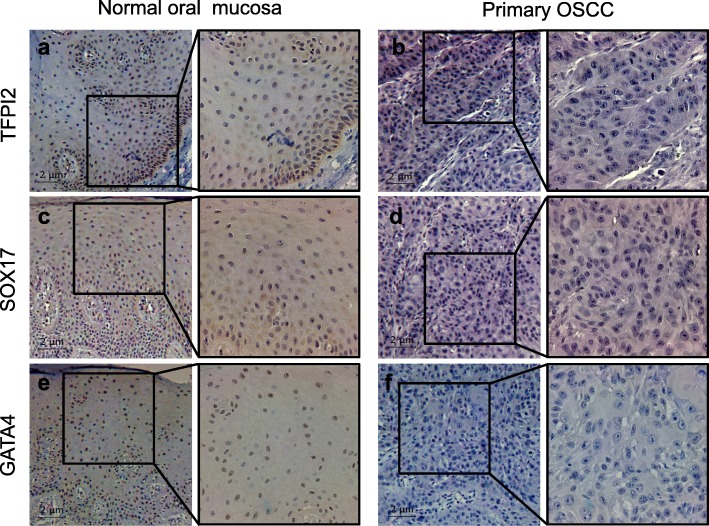
Protein expression levels of TFPI2, SOX17, and GATA4 in primary OSCC tumors and normal oral mucosa. Representative immunohistochemical analysis results showing **a**, **b** TFPI2, **c**, **d** SOX17, and **e**, **f** GATA4 expression in primary OSCC tissues and normal oral mucosa (right images: × 100; left images: × 400)

Next, we examined the protein expression of the three genes by immunohistochemical staining. Normal oral mucosal tissue showed strong nuclear positivity against TFPI2, SOX17, and GATA4 as well as cytoplasmic staining for TFPI2 and SOX17 (Fig. 5, Additional file 1: Figure S2). In contrast, those proteins were negatively expressed in both the nucleus and cytoplasm of the primary OSCC tissue.

### Methylation of *TFPI2*, *SOX17*, and *GATA4* is associated with OSCC patient survival

**Fig. 6:**
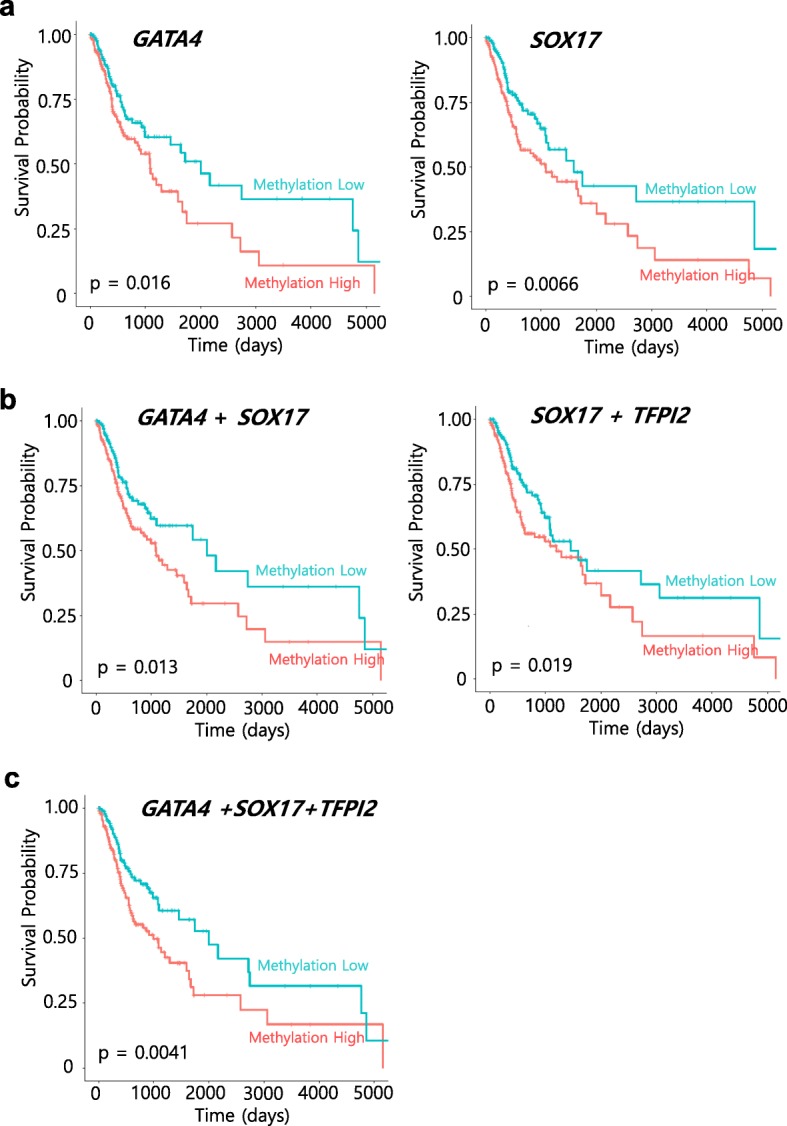
Kaplan–Meier survival curves for the 344 patients with OSCC from TCGA HNSCC cohort according to the methylation status of the three candidate genes. Overall survival for **a** *GATA4* (cg24910352 located on gene body) and *SOX17* (cg04672706 located on promoter region), respectively. **b** Combination of *GATA4* and *SOX17* genes methylation (left) and *TFPI2* (cg22441533 located on promoter region) and *SOX17* genes methylation (right). **c** Combination of *TFPI2*, *GATA4*, and *SOX17* genes methylation. Probe positions were annotated using the UCSC genome browser. Clinical information of patients with OSCC (*n* = 344) were divided into two groups (methylation high group; orange line, *n* = 172; methylation low group; blue line, *n* = 172) according to their methylation status (*β*-values) on *TFPI2*, *SOX17*, or *GATA4*. A probability of <  0.05 (log rank test *p < 0.05) was considered to represent a statistically significant difference

To investigate whether the methylation status of the three potential biomarkers (*TFPI2*, *SOX17*, and *GATA4*) described above had an impact on overall survival of OSCC patients, TCGA DNA methylation data (CpG sites) with clinical information (*n* = 344) were divided into two groups (hypermethylated group, *n* = 172; hypomethylated group, *n* = 172) according to their methylation status on *TFPI2*, *SOX17*, or *GATA4*. Kaplan–Meier analysis was performed on either each CpG on the promoter region or combinations of CpGs. To explore the potential clinical significance of these data, we tested these above three tumor suppressor genes for their prognostic potential in a large TCGA cohort (head and neck squamous cell carcinoma; HNSCC) [15]. We subtracted the patients with OSCC from HNSCC TCGA cohort and well-annotated clinical data that could be correlated with survival and gene methylation status. Strikingly, we observed a statistically significant increased risk for mortality when either individual genes or combination of genes were methylated. For example, Kaplan–Meier survival curves for *GATA4* and *SOX17*, individually, show that DNA methylation of each is associated with decreased survival but no significant statistical results for *TFPI2* alone (Fig. 6a, *p* = 0.016 for *GATA4*, *p* = 0.0066 for *SOX17*), even though TPFI2 gene was frequently hypermethylated in both OSCC cells and OSCC primary tumors. We wondered whether the combination of two- or three-gene methylation could have an impact on overall survival in this cohort. Very interestingly, combination of two-gene (*SOX17* and *GATA4*) methylation is associated with significantly worse survival (Fig. 6b, *p* = 0.013). In addition, *SOX17* combination of *TFPI2* gene methylation is associated with worse survival as well (Fig, 6)b, *p* = 0.019, but there are no statistically significant results for combination of *GATA4* and *TFPI2* genes. More importantly, Kaplan–Meier survival curves for *TFPI2*, *GATA4*, and *SOX17* show that DNA methylation of three-gene combination is associated with significantly worse survival (Fig. 6c, *p* = 0.0041). These findings support our hypothesis that DNA methylation of TSGs may have an important role during OSCC cancer progression as well as clinical application as prognostic biomarkers.

## Discussion

The aberrant promoter hypermethylation in the adjacent of transcription start sites (TSSs) often leads to alterations in gene function and abnormal cellular pathway in human cancer. Epigenetic events linked to TSG inactivation through promoter methylation are as frequent as somatic mutations in cancer and contribute to drive tumor initiation and progression. The promoter methylation of 14 selected TSGs in this study has previously been identified in colon cancer [3, 16] and has been characterized in other cancer types [4]. Recently, it has been demonstrated that OSCC involves genetic alterations and epigenetic mechanisms play crucial roles [17]. Epigenetically regulated genes have become an important tool for better understanding cancer initiation, progression, and development.

Interestingly, recent studies have demonstrated that *Fusobacterium nucleatum* (Fn), a specific species of the gut microbiota, may be closely associated with colorectal carcinogenesis [18]. A growing body of evidence suggests that Fn promotes tumor progression and inhibits the antitumor immune response in the colorectum by regulating the β-catenin pathway [8, 18]. In addition, Fn DNA is significantly enriched in the microsatellite instability-high (MSI-H) molecular subtypes of CRC samples [19, 20]. These studies encouraged us to investigate whether the epigenome profile of colon cancer might be shared with OSCC. We therefore tested the hypothesis that selected TSGs, which are frequently hypermethylated in colon cancer, predominantly promote methylation in OSCC tumors. We observed interesting relationships among the most methylated genes (*TFPI2*, *SOX17*, and *GATA4*) in OSCC cell lines and primary tumors in a cancer-specific manner.

Tissue factor pathway inhibitor-2 (*TFPI2*), a Kunitz-type serine proteinase inhibitor, can inhibit a variety of serine proteases, including factor VIIa/tissue factor, factor Xa, plasmin, trypsin, chymotrypsin, and plasma kallikrein. Over the last decade, *TFPI2* has been identified as a TSG in several types of cancer, including colorectal cancer (CRC) [21, 22]. *TFPI2* methylation frequently existed in CRC patients’ sera [23] and stool samples [21]. Moreover, hypermethylated *TFPI2* was associated with recurrence and early-stage CRC [24], and *TFPI2* was significant in CRC patients’ sera with large, poorly differentiated carcinoma, deep invasion, lymph node metastasis, or distant metastasis [23]. Although the methylation of *TFPI2* in OSCC has been identified using a genome-wide methylation array, its promoter hypermethylation associated with transcriptional silencing was not validated in OSCC tumor samples [25]. In this study, we validated that *TFPI2* is frequently methylated in a panel of OSCC primary tumor samples in a cancer-specific manner and that *TFPI2* is transcriptionally regulated by promoter hypermethylation in OSCC cells. Our study thus highlights the finding that *TFPI2* is hypermethylated in most OSCC tumor samples we tested in this study, suggesting that *TFPI2* may be a useful biomarker for screening OSCC patients. Therefore, further studies in a larger series of samples will be necessary to confirm that the *TFPI2* gene can be a useful methylation biomarker for screening OSCC patients.

*SOX17* encodes an HMG box transcription factor and has been implicated in oligodendrocyte development, vascular development, the formation of definitive endoderm, and embryonic hematopoiesis [26–29]. The *SOX17* promoter is hypermethylated in cholangiocarcinoma (CCA) tissues, lung cancer [30], gastric cancer [31], liver cancer [32], and breast cancer [33]. Additionally, epigenetically regulated *SOX17* may contribute to the abnormal activation of the Wnt signaling pathway in human cancer [34]. The high frequency of *SOX17* methylation in 50% of non-small-cell lung cancers and nearly 90% of esophageal squamous cancers strongly supports this possibility and is consistent with the known role of the aberrant activation of Wnt signaling during tumorigenesis for multiple cancer types [35].

The *GATA4* gene encodes a member of the GATA family of zinc-finger transcription factors, which recognize the GATA motif present in the promoters of many genes. Transcription factors GATA4 play an essential role in the development and differentiation of the gastrointestinal tract and are suggested to be involved in colorectal cancer development [36, 37]. *GATA4* is known to be more likely to behave as a tumor suppressor gene since the increased expression levels correlate with the terminal differentiation in the intestinal epithelium and the terminal differentiation induced in CRC cells by sodium butyrate [36].

The loss of *GATA4* expression due to promoter hypermethylation has been reported in primary colorectal, gastric, esophageal, lung, and ovarian cancers [37–40]. To our knowledge, this is the first study to describe how the promoter hypermethylation of both the *SOX17* and *GATA4* genes is regulated in OSCC cells and how these promoters are frequently hypermethylated in OSCC primary tumors.

Epigenetic alterations of specific genes have recently emerged as potential candidate biomarkers for the early detection of cancer [1]. Recent advances attest to the great promise of DNA methylation markers as powerful tools for future use in the clinic. It would be of great value to find useful biomarkers and prognostic molecular signatures in OSCC to develop novel therapeutic strategies or chemotherapeutic agents. Numerous potential clinical applications of epigenetics for diagnostic and therapeutic applications have been reported [41].

The promoter hypermethylation of specific genes has already been studied in oral premalignancy [42, 43], but little is known about the description of early DNA methylation changes during oral carcinogenesis using genome-wide profiles. Recently, several studies have reported that early DNA promoter methylation alterations are associated with histological changes during oral tumorigenesis [44]. They confirmed that these alterations are early events during oral tumorigenesis and offer an opportunity for biomarker development [45].

According to our KM analysis for patients with OSCC from TCGA data, we found a statistically significant increased risk for mortality when either individual genes or combination of genes were methylated. Unlikely *SOX17* and *GATA4*, we did not see any significant clinical correlation with overall survival of *TFPI2* alone. Promoter methylation of *TFPI2* is detected in most of tumors (over 90% tested cancer patients samples) with OSCC and other cancer types [21] as well which explained *TFPI2* methylation can be useful for early detection biomarker rather than prognostic biomarkers. Previous studies have reported survival-related OSCC methylation biomarkers [46, 47]. These reports described novel methylation biomarkers associated with prognostic biomarkers in OSCC, showing that the methylation frequency of candidate genes ranged from 10 to 65%. However, *TFPI2* and *SOX17* in our study showed a significant hypermethylation pattern (95% and 67%, respectively) in OSCC primary tumors compared to those observed in previous studies because we used a well-established validation approach using a specific promoter region to identify methylation biomarker candidates. Based on our data, the dysregulation of the *TFPI2*, *SOX17*, and *GATA4* genes regulated by epigenetic changes may explain a main mechanism linked to OSCC development, and these three-gene panels could serve as synergistic prognostic biomarkers in OSCC treatment. Further studies are needed to verify *TFPI2* and *SOX17* as methylation biomarkers in a large cohort of OSCC primary tumors along with normal controls, and these genes should be evaluated by testing in blood or sputum DNA.

## Conclusions

In summary, we used 14 putative TSGs originally identified in colon cancer to screen the epigenetic regulation of these genes in 10 OSCC cell lines, a small set of primary OSCC tumors, and normal oral mucosa. We identified the transcriptional expression of 9 genes regulated by promoter hypermethylation in OSCC and finally provided 3 genes (*TFPI2*, *SOX17*, and *GATA4*) that were frequently hypermethylated in primary OSCC tumors in a cancer-specific manner. The methylation level in the promoter region of these genes was confirmed by bisulfite genomic sequencing analysis, which suggested that the methylation level is significantly increased in OSCC cell lines and primary tumors compared to the methylation level in normal tissues. Our study thus highlights the finding that the dysregulation of *TFPI2*, *SOX17*, and *GATA4* genes regulated by epigenetic changes may explain a main mechanism linked to OSCC development, and this three-gene panel has a potential to be used as a synergistic biomarker set capable of improving the prognosis and treatment for patients with OSCC.

## Methods

### Tissue samples

For OSCC samples, paraffin-embedded tissue specimens from patients who were diagnosed and surgically treated at the Department of Oral and Maxillofacial Surgery, Pusan National University Dental Hospital, between 2009 and 2014 were retrieved. For normal oral mucosal tissue for comparison, fresh healthy tissue that had been obtained during dental procedures, such as gingivoplasty, third molar extraction, or implant surgery, was used. Clinicopathologic features, including age, gender, histopathologic grade, and the TNM stage, are summarized in Table 1. Tumor grading was based on the ethical permission for this study and was granted by the Institutional Review Board (IRB) of Pusan National University Dental Hospital (IRB No. PNUDH-2019-006).

### Cell culture and 5-aza-dC treatment

Ten human OSCC cell lines (Ca9-22, HSC4, OSC20, SAS, SCC25, HN22, YD10B, YD38, YD9, and HSC3) were used in this study. The Ca9-22 and HSC4 cell lines were cultured in MEM/EBSS (HyClone, Logan, UT) medium. The OSC20, SAS, and SCC25 cell lines were cultured in a 1:1 mixture of Dulbecco’s modified Eagle’s medium and Ham’s F-12 Nutrient Mixture (DMEM/F12; HyClone, Logan, UT). The HN22, YD10B, YD38, YD9, and HSC3 cell lines were cultured in a 1:1 mixture of Dulbecco’s modified Eagle’s medium (HyClone, Logan, UT). All cell culture medium was supplemented with 10% fetal bovine serum (HyClone, Logan, UT, USA) and 1% antibiotic–antimycotic (Gibco, Grand Island, NY, USA). All cell lines were incubated at 37 °C in atmospheric conditions of 20% O_2_ and 5% CO_2_. To investigate the effects of 5-aza-dC treatment, the cells were treated with 5 μM 5-aza-dC (Sigma, St. Louis, MO, USA) for 72 h.

### DNA methylation analysis

For methylation analyses, genomic DNA was isolated from 10 OSCC cell lines, 33 primary OSCC tissues, and 11 normal oral mucosa samples using phenol/chloroform. The bisulfite modification of 2 μg genomic DNA was performed using the EZ DNA Methylation Kit (Zymo Research, CA, USA). For positive and negative controls, in vitro methylated DNA (IVD) and H_2_O were used. Primer pairs for methylation analyses were preferentially designed for CpG islands of the target genes [21, 34, 37, 48]. The primer sequences are listed in Additional file 2: Table S1. Methylation analyses, including MSP and bisulfite sequencing analysis, were performed as previously described [49].

### Bisulfite sequencing

One microgram of genomic DNA from each sample was bisulfite converted using the EZ DNA Methylation Kit (Zymo Research, CA, USA) following the manufacturer’s protocol. The PCR conditions and primer sequences are provided in Additional file 2: Table S1. The PCR amplicons were gel-purified and subcloned into the pCRII-TOPO vector (Invitrogen, Carlsbad, CA). At least five to seven clones were randomly selected and sequenced on an ABI3730xl DNA analyzer to ascertain the methylation patterns of each locus.

### Quantitative real-time RT-PCR

Total RNA was isolated from the human normal colon and UC patient tissues using TRI-Solution (Bio Science Technology) following the manufacturer’s protocol. RNA quantity was measured using a NanoDrop 2000/2000c instrument (Thermo Scientific, MA, USA), and 1 μg of total RNA was reverse-transcribed into cDNA using the iScript^TM^cDNA Synthesis Kit (Bio-Rad). For expression studies, the primers are listed in Additional file 2: Table S1. Quantitative RT-PCR was performed on a CFX96^TM^ Real-Time PCR Detection System (Bio-Rad) using SYBR Green Master Mix (Thermo Scientific, MA, USA). The expression levels of the target genes were normalized to the *GAPDH* levels, and all the relative quantifications of expression were calculated using the 2^−ΔΔCt^ method.

### Immunohistochemistry (IHC)

Formalin-fixed, paraffin-embedded specimens of OSCC and normal oral mucosal tissues were sectioned to a 5-μm thickness. The sections were deparaffinized in xylene and rehydrated through graded alcohol solutions. Heat-induced antigen retrieval was performed in a microwave oven for 10 min in Tris/EDTA buffer (pH 9.0). Endogenous peroxidase activity was inactivated by treating with 3% H_2_O_2_ in PBS for 15 min. After blocking nonspecific binding (0.75% BSA in PBS), the sections were incubated with the anti-TFPI2 (Abcam, dilution 1:100), anti-SOX17 (Abcam, dilution 1:100), and anti-GATA4 antibodies (Abcam, dilution 1:100) overnight at 4 °C. The staining was visualized by a peroxidase-conjugated secondary antibody and diaminobenzidine (Vector labs, Burlingame, CA, USA). Finally, the sections were counterstained by Mayer’s hematoxylin and were mounted and photographed with an Axioplan microscope (Carl Zeiss, Germany). Primary antibodies were omitted for the negative controls.

### Kaplan–Meier survival analysis using TCGA DNA methylation data

Head and neck squamous cell carcinoma (HNSCC) DNA methylation dataset (HumanMethylation 450 k Illumina; *n* = 528) in The Cancer Genome Atlas (TCGA) database [15] was initially downloaded from the firebrowse website (http://firebrowse.org/). Then, samples related to OSCC (*n* = 344) were selected for survival analysis from HNSCC TCGA cohort (patients (*n* = 184) from tonsil, larynx, oropharynx, hypopharynx, and lip were excluded based on anatomic subdivision). Patients were divided into two groups (high—hypermethylated, *n* = 174; low—hypomethylated, *n* = 174) according to the methylation level (*β* value) on each CpG site located in the promoter regions of *TFPI2* and *SOX17*, but in gene body region of *GATA4*. The R package survminer (https://github.com/kassambara/survminer) was used for the analysis. *P* values were calculated using the log-rank test.

### Statistical analysis

Quantified data are expressed as the mean ± standard deviation (SD). Significance testing was conducted using Student’s *t* test.

## Additional files

Additional file 1: **Figure S1.** Correlation between transcriptional expression by qRT-PCR and the promoter hypermethylation of tumor suppressor genes by MSP analysis in OSCC cell lines. Related to Fig. 1a. **Figure S2.** Protein expression levels of TFPI2, SOX17, and GATA4 in additional set of primary OSCC tumors and normal oral mucosa. Related to Fig. 5. (PPTM 3917 kb)

Additional file 2: **Table S1.** Selected gene primers for MSP, bisulfite sequencing, and RT-PCR analyses. (DOCX 13 kb)

## Data Availability

Not applicable.

## Abbreviations

5-aza-dC: 5-Aza-2′-deoxycytidine
Fn: Fusobacterium nucleatum
IHC: Immunohistochemistry
KM survival curve: Kaplan–Meier survival curves
MSP: Methylation-specific PCR
OSCC: Oral squamous cell carcinoma
RT-PCR: Reverse transcriptase polymerase chain reaction
TSGs: Tumor suppressor genes
TSSs: Transcription start sites
