# Supplementary material for: Aberrantly hypermethylated tumor suppressor genes were identified in oral squamous cell carcinoma (OSCC)
Source: Clin Epigenetics. 2019 Aug 12;11:116. doi: 10.1186/s13148-019-0715-0 (PMC6689875; doi:10.1186/s13148-019-0715-0)
Supplement: Supplementary file 1 — Figure S1. Correlation between transcriptional expression by qRT-PCR and the promoter hypermethylation of tumor suppressor genes by MSP analysis in OSCC cell lines. Related to Fig. 1a. Figure S2. Protein expression levels of TFPI2, SOX17, and GATA4 in additional set of primary OSCC tumors and normal oral mucosa. Related to Fig. 5. (PPTM 3917 kb) [file 13148_2019_715_MOESM1_ESM.pptm]

## Slide 1
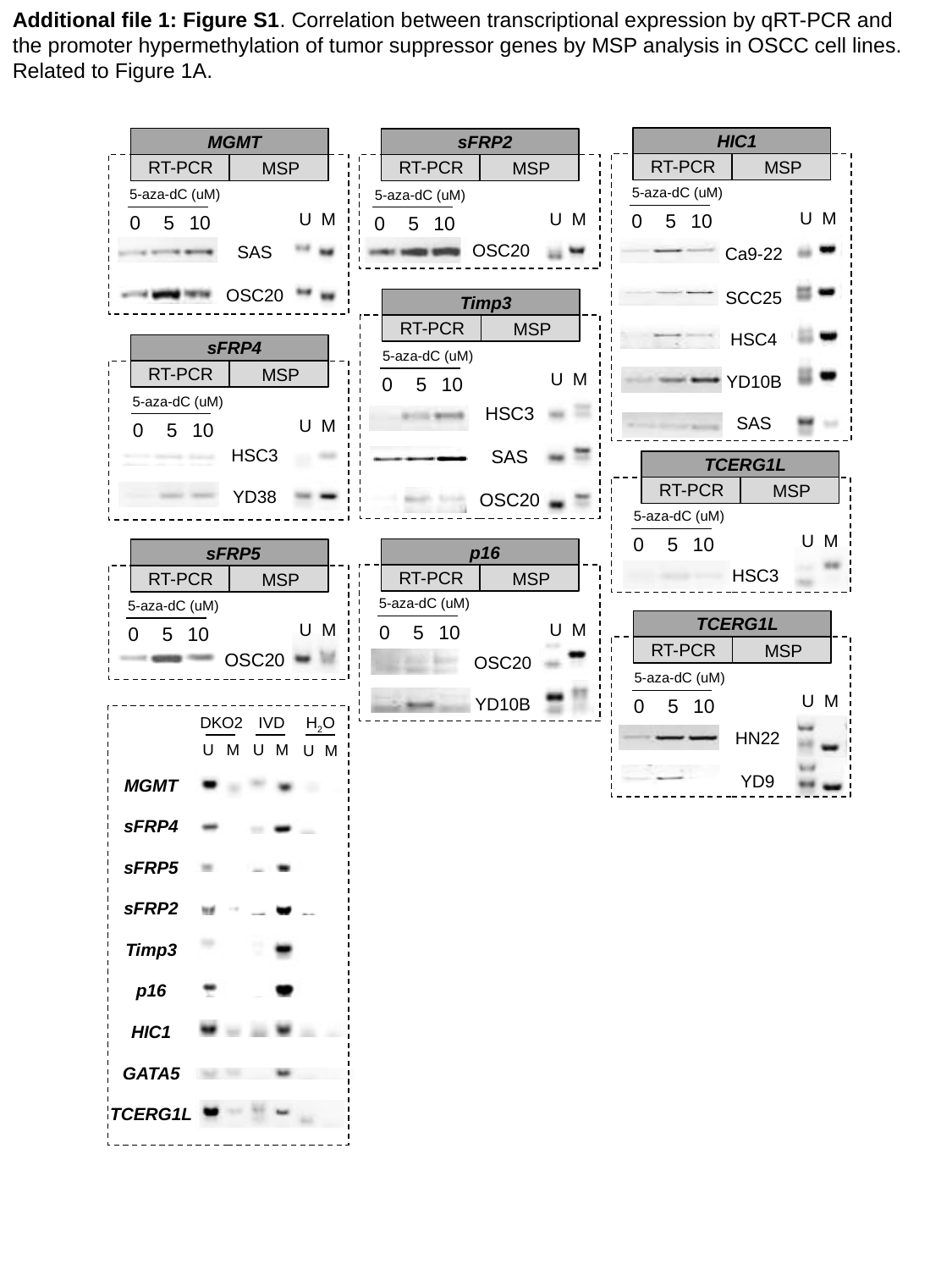

Additional file 1: Figure S1. Correlation between transcriptional expression by qRT-PCR and the promoter hypermethylation of tumor suppressor genes by MSP analysis in OSCC cell lines. Related to Figure 1A.
HIC1
RT-PCR
MSP
5-aza-dC (uM)
U
M
0
5
10
Ca9-22
SCC25
HSC4
YD10B
SAS
MGMT
RT-PCR
MSP
5-aza-dC (uM)
U
M
0
5
10
SAS
OSC20
sFRP2
RT-PCR
MSP
5-aza-dC (uM)
U
M
0
5
10
OSC20
Timp3
RT-PCR
MSP
5-aza-dC (uM)
U
M
0
5
10
HSC3
SAS
OSC20
sFRP4
RT-PCR
MSP
5-aza-dC (uM)
U
M
0
5
10
HSC3
YD38
TCERG1L
RT-PCR
MSP
5-aza-dC (uM)
U
M
0
5
10
HSC3
p16
RT-PCR
MSP
5-aza-dC (uM)
U
M
0
5
10
OSC20
YD10B
sFRP5
RT-PCR
MSP
5-aza-dC (uM)
U
M
0
5
10
OSC20
TCERG1L
RT-PCR
MSP
5-aza-dC (uM)
U
M
0
5
10
HN22
YD9
DKO2
IVD
H2O
U
M
U
M
U
M
MGMT
sFRP4
sFRP5
sFRP2
Timp3
p16
HIC1
GATA5
TCERG1L

## Slide 2
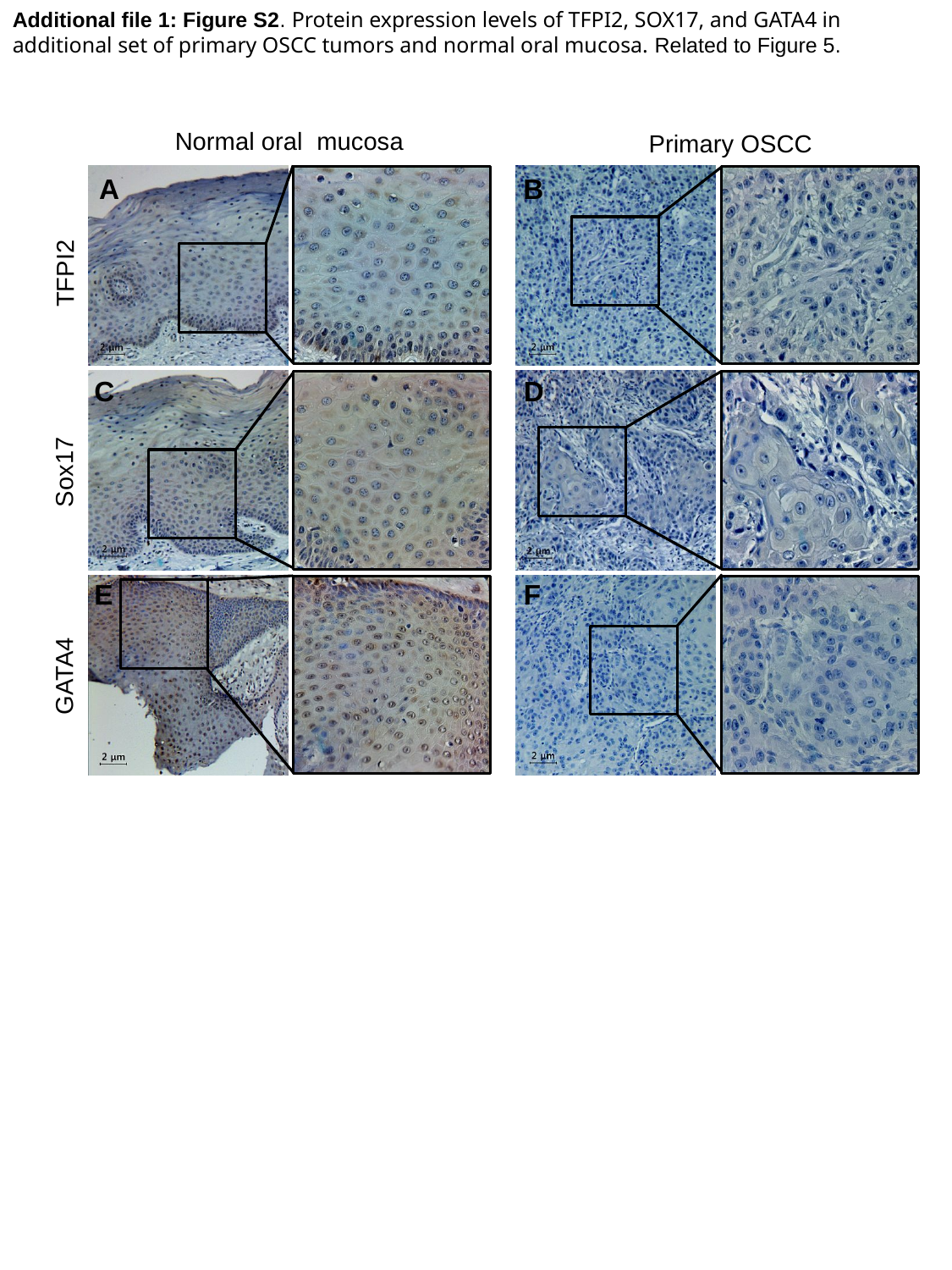

Additional file 1: Figure S2. Protein expression levels of TFPI2, SOX17, and GATA4 in additional set of primary OSCC tumors and normal oral mucosa. Related to Figure 5.
Normal oral mucosa
Primary OSCC
A
B
TFPI2
C
D
Sox17
E
F
GATA4
