# Supplementary material for: Aberrantly hypermethylated tumor suppressor genes were identified in oral squamous cell carcinoma (OSCC)
Source: Clin Epigenetics. 2019 Aug 12;11:116. doi: 10.1186/s13148-019-0715-0 (PMC6689875; doi:10.1186/s13148-019-0715-0)
Supplement: Supplementary file 2 — Table S1. Selected gene primers for MSP, bisulfite sequencing, and RT-PCR analyses. (DOCX 13 kb) [file 13148_2019_715_MOESM2_ESM.docx]

| **Gene** | **Target** | **Methylation specific PCR** | |
| --- | --- | --- | --- |
|  |  | **Forward primer (5’-3’)** | **Reverse primer (5’-3’)** |
| *TFPI2* | Unmethylation | CCCACATAAAACAAACACCCAAACCA | TGGTTTGTTGGGTAAGGTGTTTG |
|  | Methylation | CATAAAACGAACACCCGAACCG | GTTCGTTGGGTAAGGCGTTC |
| *FBN2* | Unmethylation | GTTTTGTTGGGTTTTTAAAATTTTTGTGTTGTG | AAATAACAACTACAAAACCAAACAAAAATACA |
|  | Methylation | GGGTTTTTAAAATTTTCGCGTCGC | CTACGAAACCGAACGAAAATACG |
| *GATA4* | Unmethylation | TTTGTATAGTTTTGTAGTTTGTGTTTAGT | CCCAACTCACAACTCAAATCCCCA |
|  | Methylation | GTATAGTTTCGTAGTTTGCGTTTAGC | AACTCGCGACTCGAATCCCCG |
| *Sox17* | Unmethylation | GTTTTGTTGGGTTTTTAAAATTTTTGTGTTGTG | AAATAACAACTACAAAACCAAACAAAAATACA |
|  | Methylation | GGGTTTTTAAAATTTTCGCGTCGC | CTACGAAACCGAACGAAAATACG |
| **Gene** | **Locus** | **Bisulfite sequencing** | |
|  |  | **Forward primer (5’-3’)** | **Reverse primer (5’-3’)** |
| *TFPI2* |  | GGTTTATGGTGTAGGGG | CAATCACTAACAAATCATTTCC |
| *FBN2* |  | ATATGAAGGTGAAGGGCGAGG | CTACACACCCCTAATTTTAAAC |
| *GATA4* |  | CTTCCAACCCYACCTTC | GTTTTTAGAAGAAGAGGAGGG |
| *SOX17* |  | TAATAAAGTTGATTTTGGGTATTATAG | CCCTACCTACTAAACCTAAAAATTC |
| **Gene** | | **Quantitative real-time PCR** | |
|  |  | **Forward primer (5’-3’)** | **Reverse primer (5’-3’)** |
| *TFPI2* | | GGGCCCTACTTCTCCGTTAC | CACACTGGTCGTCCACACTC |
| *FBN2* | | AGGGATCCTTCATTTGCCAC | GCCCGGGGTATTTACACACT |
| *GATA4* | | GGCCGCCCGACACCCCAATCT | ATAGTGACCCGTCCCATCTCG |
| *SOX17* | | CAGAATCCAGACCTGCACAA | GCGGCCGGTACTTGTAGTT |

**Table S1.** Selected gene primers for MSP, Bisulfite sequencing, RT-PCR analyses
